# Successful Treatment of Depressed, Distensible Acne Scars Using Autologous Fibroblasts: A Multi-Site, Prospective, Double Blind, Placebo-Controlled Clinical Trial

**DOI:** 10.1111/dsu.12204

**Published:** 2013-04-08

**Authors:** Girish S Munavalli, Stacy Smith, John M Maslowski, Robert A Weiss

**Affiliations:** * Dermatology, Laser & Vein Specialists of the Carolinas, Charlotte, North Carolina and Department of Dermatology, Wake Forest UniversityWinston Salem, NC; † Private PracticeDelMar, California; ‡ Fibrocell Technologies Inc.Exton, Pennsylvania; § Maryland Laser, Skin and Vein InstituteHunt Valley, Maryland

## Abstract

**Background** A previous clinical trial evaluating autologous fibroblasts (human dermal) injections for the treatment of facial contour deformities found significantly greater improvements in wrinkle and acne scar appearance than with placebo treatment.

**Objective** To compare the efficacy and safety of autologous fibroblast treatment of moderate to severe, depressed, distensible facial acne scars with that of vehicle control.

**Methods** This was a randomized multicenter, double-blind, placebo-controlled trial in subjects with bilateral moderate to severe acne scarring; subjects served as their own controls. Skin biopsies were obtained from randomized subjects for fibroblast production. Subjects (*n* = 99) underwent three intradermal injection sessions with 2 mL of autologous fibroblast suspension (10–20 million cells/mL) on one cheek and vehicle control (cell culture medium) on the other at 14-day intervals. Efficacy was based on the blinded subject’s, evaluator’s, and independent photographic viewer’s (IPR) assessment of acne scarring 1 to 4 months after the last treatment.

**Results** Autologous fibroblast treatment was associated with significantly greater treatment success than vehicle control for the subject (43% vs 18%), evaluator (59% vs 42%), and IPR assessments. Autologous fibroblast injections were well tolerated, without permanent adverse effects.

**Conclusions** Autologous fibroblast injections safely and effectively improved the appearance of depressed distensible acne scars.

Of all the skin changes possible after inflammatory and nodulocystic acne eruptions of the face, including postacne erythema, dyschromia, and scarring, scarring leaves the most potentially permanent, cosmetically and psychologically devastating effects.^1^ Active acne, and probably facial acne scarring, is associated with negative psychosocial effects and poor quality of life.^1^ Acne scarring may not resolve spontaneously, and procedural interventions including surgery are required to reverse these skin changes. Acne scarring can be classified morphologically into atrophic or hypertrophic, or more specifically boxcar or fixed, icepick, and distensible or rolling scars.^2^,^3^ As expected given the diversity of acne scarring morphology and severity, certain treatment modalities are more effective with certain subgroups of scarring.^4^ For example, distensible scars are amenable to volumetric correction with dermal fillers. Even with the development of new treatment options, multiple treatment modalities are likely to be required to treat acne-scarred individuals.^5^–^6^

The advent of dermal fillers began with bovine collagen usage in the 1980s. In a small series, bovine collagen was shown to have efficacy in the treatment of acne scarring.^7^ Several studies have characterized the effectiveness and longevity of different types of permanent and semipermanent dermal fillers in the treatment of acne scarring.^8^,^9^ Such fillers include hyaluronic acid (HA)-based products with varying degrees of cross-linking. These HA products have themselves been shown to stimulate endogenous collagen formation over time, which could contribute to sustained volumetric correction of treated scars,^11^ but the use of dermal fillers is not without risk,^12^ and an awareness of existing treatment algorithms is needed to manage potential complications.^13^ Classically, device treatments such as dermabrasion and laser resurfacing have been considered first-line treatment of atrophic acne scarring.^2^–^14^ Most recently, fractional carbon dioxide (CO_2_) ablative laser was used successfully to treat atrophic acne scarring in a small randomized controlled, blinded evaluation.^15^ Subjects received three treatments at 4- to 5-week intervals. The authors concluded that acne scars can be safely improved using ablative fractional CO_2_ laser resurfacing, with improvement seen as soon as after 1 month and sustained 6 months after treatment. They noted that the use of higher energy levels might have improved the results and possibly induced significant adverse effects.^15^

The purpose of the study was to compare the safety and efficacy profile of autologous fibroblast treatments (LaViv, azficel-T, Fibrocell Sciences, Inc, Exton, PA) of moderate to severe depressed, distensible acne scars with that of vehicle control treatments.

## Materials and Methods

A centralized Institutional Review Board, Chesapeake Research Review, Inc., reviewed and approved the protocol and informed consent forms, and written informed consent was obtained from all 99 subjects at seven U.S. sites before study participation. The study was conducted in accordance with Good Clinical Practices and principles that have their origins in the Declaration of Helsinki.

**Figure 1:**
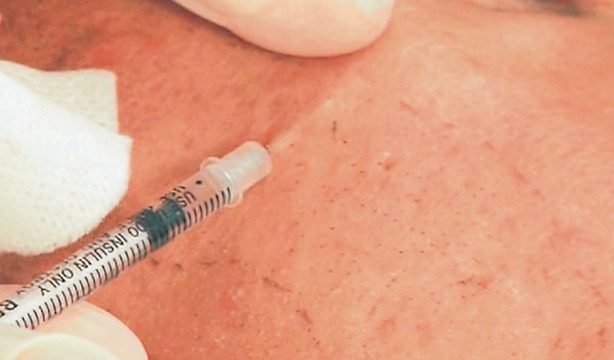
Treatment was performed using a 28 G tuberculin syringe. Note the superficial nature of the needle placement, below and surrounding each identifiable scar in the field. The endpoint of this injection is a visible wheal with blanching (276 × 162 mm; 72 × 72 dots per inch).

Depressed, distensible facial acne scars (scars disappearing completely with manual perilesional skin stretching) were targeted for treatment in this study. Evaluators were provided with a pictorial and descriptive guideline outlining the acne scarring morphology (depressed and distensible) considered for autologous fibroblast treatment.^16^ Healthy subjects with facial acne scarring on both cheeks were enrolled in the study. To meet eligibility, subjects’ depressed distensible acne scars on both cheeks had to be evaluator rated as moderate or severe on a novel, validated 5-point acne scar assessment scale (Table 1). Subject ratings of the appearance of each cheek were required to be very dissatisfied or dissatisfied (Table 2). Subjects were excluded if they had hypertrophic acne scarring or numerous icepick acne scars in the treatment area, had undergone aesthetic procedures (e.g., fractional or traditional ablative/non-ablative laser resurfacing, subcision, microdermabrasion, chemical peels) to the treated area within the past 12 months, or had ever previously received dermal fillers in the treated area. Subjects with a history of heavy smoking, alcohol or drug abuse, or steroid treatment were excluded because these attributes may be associated with limited cell expansion in vitro.^17^ Prescription topical treatments, such as retinoids or topical antibiotics, were discontinued for 2 weeks before the first injection and disallowed for the duration of the study. Enrolled subjects underwent three postauricular full-thickness (epidermis, dermis, fat) skin punch biopsies to harvest autologous fibroblasts. The cosmetically inconspicuous postauricular mastoid area was chosen because of its sun-protected nature. Biopsy sites were closed using 5–0 fast-absorbing chromic gut suture or with thin adhesive strips. The 3-mm punch biopsy specimens (times 3) were immediately placed into a sterile vial containing transport medium. The vials were then transported in a biocontainer with ice packs on the day of harvest overnight to Fibrocell Technologies, Inc. (Exton, PA), where the fibroblasts were isolated, cultured, and expanded over a several-week period as described previously.^18^ In a split-face design, the cheeks of each subject were randomized to receive autologous fibroblasts (10–20 million cells/mL) or vehicle control (dye-free, protein-free cell culture medium) injected into the high papillary dermis at a maximum dose of 2 mL per treatment, administered as approximately 0.1 mL/cm^2^, with a minimum treatment area of 9 cm^2^ (Figure 1). Immediately before the injections, the treatment area on the cheek was anesthetized with topical lidocaine anaesthetic cream for 30 to 60 minutes. Most commonly, 4% lidocaine cream was employed, although some sites used a compounded formulation of benzocaine 20%, tetracaine 10%, and lidocaine 4%. The injections were made into the papillary dermal plane, using an insulin hub-less syringe with a 28G needle (Becton, Dickinson and Company, Franklin Lakes, NJ) so as to create a wheal and transient blanching of the skin surface with each injection. Injections were directed underneath the individual scar lesions, in addition to the perilesional area, to achieve a “field” treatment effect. The intent of injection was to introduce the live cells into the scarred area and surrounding skin within the confines of the papillary dermis, with the assumption that the cells would disperse into the surrounding dermis. Achieving a degree of correction, as commonly employed in techniques with dermal fillers (e.g., full correction or overcorrection), was not the endpoint, because the liquid cell suspension was not designed for immediate volumetric improvement. Wheals created at each injection point usually disappeared within hours after injection. After injection, small ice packs were used to reduce any stinging or discomfort, and subjects were instructed on a strict 7-day post-treatment regimen, including the use of only bland moisturizer and sunscreen. The use of make-up or topical cosmeceuticals was not allowed for 1 week after treatment. Subjects received three treatments to each side of the face 14 ± 3 days apart.

**Table 1:** Validated Physician Evaluator 5-point Acne Scar Assessment Scale

Grade	Term	Description
0	Clear	No depressions are seen in the treatment area. Macular discoloration may be seen
1	Very mild	A single depression is easily noticeable with direct lighting (deep). Most or all of the depressions seen are only readily apparent with tangential lighting (shallow)
2	Mild	A few to several but less than half of all the depressions are easily noticeable with direct lighting (deep). Most of the depressions seen are only readily apparent with tangential lighting (shallow)
3	Moderate	More than half of the depressions are apparent with direct lighting (deep)
4	Severe	All or almost all the lesions can be seen with direct lighting (deep)

**Table 2:** Subject Acne Scar Self-Assessment Scale

How do you feel about the appearance of your cheek?
−2 = I am very dissatisfied with the appearance of my cheek
−1 = I am dissatisfied with the appearance of my cheek
0 = I am somewhat satisfied with the appearance of my cheek
+1 = I am satisfied with the appearance of my cheek
+2 = I am very satisfied with the appearance of my cheek

Modified from Cohen and Holmes.^19^

Efficacy and safety evaluations were performed 1, 2, 3, and 4 months after the third treatment. The co-primary efficacy end point required that subject cheeks be considered to have responded based on the subject and evaluator acne scar assessment of treatment response to be counted as a treatment success. For the subject acne scar assessment, a responding cheek was defined as a cheek with a 2-point improvement on the subject acne scar assessment. The acne scar assessment scale is a 5-point scale (very dissatisfied = –2, dissatisfied = –1, somewhat satisfied = 0, satisfied = +1, very satisfied = +2).The scale is a modification of the scale used by Cohen and Holmes 19 (Table 2).

A 5-point evaluator live acne scar assessment scale was developed and validated (0 = clear, 1 = very mild, 2 = mild, 3 = moderate, 4 = severe) for use in live assessment of subjects to detect clinically meaningful changes in acne scar severity over time. The scale was used with an associated photo guide demonstrating acne scar appearance associated with each grade. Scale evaluation studies (conducted separately from and before the interventional clinical study described here) demonstrated the validity of the scale in detecting a 1-point improvement in scar appearance. Dermatologists with experience in treating acne scars were consulted during scale development to ensure the clinical meaningfulness of a 1-point improvement.

A responding cheek was defined as a cheek with a 1-point improvement on the evaluator live acne scar assessment scale. Evaluators were blinded to the treatment each cheek had received. The study required that the physician performing the treatment injections not be the physician performing the subject evaluations to eliminate any potential for bias based on the injection.

Photographs were taken of each subject at baseline and 2, 3, and 4 months after the third treatment using photographic equipment and procedures designed to ensure reproducible positioning and lighting of subjects. An independent panel of three board-certified dermatologists assessed change from baseline in the appearance of acne scarring using a 5-point scale (–2 = much worse, –1 = worse, 0 = no change, +1 = improved, +2 = much improved). Post-treatment photographs were graded compared with baseline photographs for each cheek at each time point. The independent photographic reviewers (IPR) were blinded to the treatment each cheek had received and viewed the photographs with respect to the time of the follow-up (at 1, 2, 3, and 4 months).

The statistical analysis compared the autologous fibroblast-treated and placebo-treated sides of the face. The primary efficacy comparisons were performed separately (in different rooms) for the co-primary endpoints (subject and evaluator assessments). Success was defined as statistically significant (*p* < .05, two-sided) results for the subject and evaluator assessments. For each endpoint and time point, the McNemar paired test of proportions was used to test the null hypothesis for the subject and evaluator assessments. The Wilcoxon signed-rank test was used to assess the comparative difference in scar appearance for autologous fibroblast– and placebo-treated cheeks for each of the three independent reviewers.

Assessments of safety included the incidence of treatment-emergent adverse events (AEs), vital signs, and physical examination assessments throughout the study period.

## Results

Fifty-seven percent of subjects from whom skin was harvested to prepare study treatment (N = 119 biopsied, N = 109 in the intention-to-treat population) were female, 72% were white. Seventeen percent of the ITT population were Hispanic; subjects had a mean age of 42 (Table 3). Sixty-six percent of the subjects rated their acne scar appearance at baseline as very dissatisfied for the autologous fibroblast–treated cheeks and 72% as very dissatisfied for the vehicle-treated cheeks (Table 4); 56% of the evaluators rated acne scar appearance at baseline as moderate for the autologous fibroblast–treated cheeks and 54% as moderate for the vehicle-treated cheeks (Table 4).

**Table 3:** Subject Demographics (*N* = 109)

Age, years	
Mean (SD)	42 (11)
Range (Min, Max)	19, 65
Gender, *n* (%)	
Female	62 (57)
Male	47 (43)
Race, *n* (%)	
White	78 (72)
Hispanic	17 (16)
Black or African American	8 (7)
Asian	5 (5)
American Indian or Alaska Native	1 (1)

**Table 4:** Baseline Acne Scar Assessments According to Treatment Area (*N* = 109)

*Baseline Assessment*	*Autologous Fibroblast–Treated Cheek*	*Vehicle Control–Treated Cheek*
	N (%)		
Subject		
Very Dissatisfied (−2)	72 (66)	78 (72)
Dissatisfied (−1)	37 (34)	31 (28)
Evaluator		
Severe (4)	48 (44)	50 (46)
Moderate (3)	61 (56)	59 (54)

Based on a visual inspection, subjects and evaluators rated the acne scar appearance of each cheek prior to receipt of treatment (baseline).

Subjects were to receive three autologous fibroblast and vehicle control treatments 14 days apart. Ten of the 119 subjects received no treatment. In these cases, the cell culture could not be harvested or produced too few cells upon harvest to complete the clinical treatment regime. Cell culture performance variability is expected because of the autologous nature of the product, primarily because of the lack of a consistent starting material source. For example, low cell yields upon cell expansion can occur when inadequate biopsy specimens are submitted for a given subject, such as portions of the dermis being absent or truncated during trimming and harvesting. No laboratory error root causes or manufacturing trends were associated with lots exhibiting low yield or inability to achieve harvest. Ninety-six of the 99 subjects treated completed the series of three treatments. Of the 99 treated subjects, there were seven early study terminations. One subject withdrew consent for reasons unrelated to AEs, and six were lost to follow-up. Subjects received a mean total dose over the three treatments of 5.9 mL of autologous fibroblast to one cheek and 5.9 mL of vehicle control to the other cheek. The average treatment area was 29 cm^2^ for autologous fibroblast and 28 cm^2^ for vehicle control. The total dose per average treatment area was 0.244 to 0.246 mL/cm^2^ (∼0.08 mL/cm^2^ for each of three treatments).

### Efficacy Endpoints

**Figure 2:**
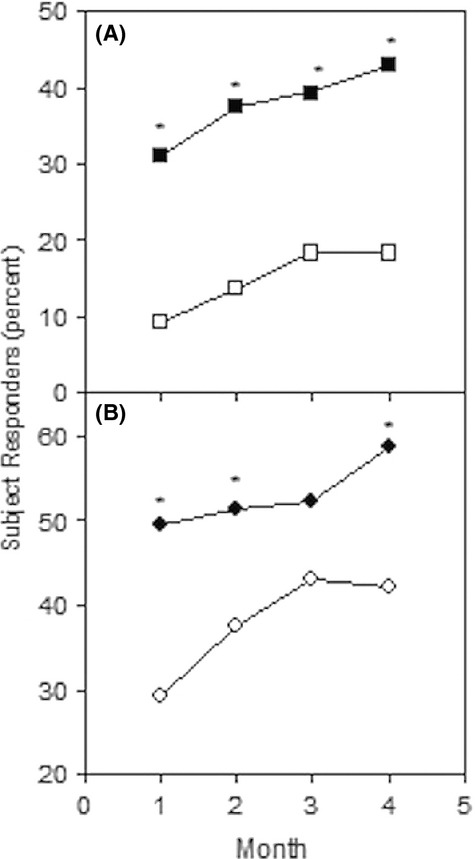
Efficacy assessments as a function of time: subjects and physician evaluators rated acne scar appearance of both cheeks 1 to 4 months after three treatments using autologous fibroblast and vehicle control as described in the Materials and Methods section. Subjects and evaluators were blinded to the treatment received on each cheek. The percentages of responders based on the subject assessment (A) are shown for autologous fibroblast– (filled squares) and vehicle control–treated (open squares) cheeks. The percentages of responders based on the evaluator assessment (B) are shown for autologous fibroblast–(filled circles) and vehicle control–treated (open circles) cheeks. A responder is defined as a 2-point improvement from baseline on the subject assessment and a 1-point improvement from baseline on the evaluator assessment. *Statistically significant difference between autologous fibroblast and vehicle control treatment based on the McNemar paired test of proportions (*p* < .05) (40 × 54 mm; 300 × 300 dots per inch).

**Figure 3:**
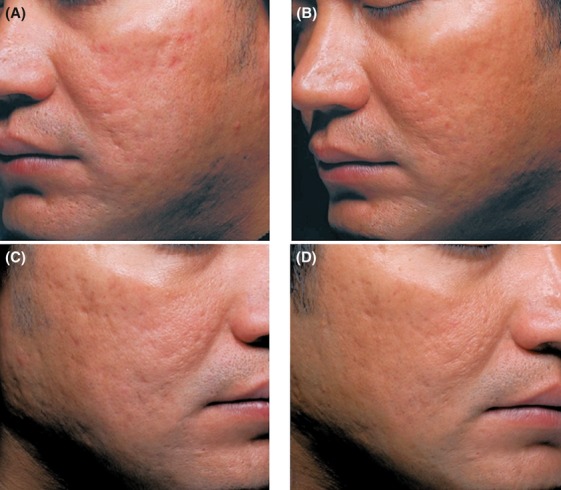
Clinical response. Subjects received three treatments on the left cheek subunit with autologous fibroblasts at 2-week intervals as described in the Materials and Methods section. Shown are sample photographs of a subject taken before (A) and 4 months after (B) treatment with autologous fibroblasts. The control side on the right cheek subunit is shown in monographs before (C) and 4 months after (D) treatment (52 × 45 mm (300 × 300 dots per inch).

Treatment with autologous fibroblast was associated with statistically significantly more responders 4 months after the last treatment for the subject and evaluator responder analysis than vehicle control (Figure 2). Co-primary endpoint *p*-values were <.001 for subject responder analysis and .01 for evaluator responder analysis. More than twice as many subjects rated the autologous fibroblast–treated area with a 2-point or greater improvement than the area receiving vehicle control (43% vs 18%). Evaluators rated 59% of the autologous fibroblast–treated sides with a 1-point or greater improvement on the evaluator scale compared with 42% of the sides receiving vehicle control. Based on subject and evaluator assessments at earlier time points, the proportion of responding autologous fibroblast treated cheeks was statistically significantly greater than that of placebo for all but one assessment at one time point (evaluator at 3 months after the last treatment). As assessed by the subject and evaluator, the response rate continued to increase throughout the follow-up period for autologous fibroblast–treated cheeks but did not increase after the 3-month visit for vehicle control–treated cheeks (Figure 2). Control and treatment side photographs from a representative subject are shown in Figure 3.

Based on the three IPR scores at three time points, all three reviewers consistently ranked the autologous fibroblast–treated cheeks as statistically significantly more improved than the placebo-control treated cheeks for all but one assessment (reviewer 2 at month 4). In all assessments, the mean score for the autologous fibroblast–treated cheeks was greater than that for the placebo-treated cheek.

### Safety Endpoints

Autologous fibroblast treatment was considered to be safe and well tolerated in this study. No subjects experienced serious AEs, discontinued treatment, or withdrew from the study as a result of a treatment-emergent AE. All AEs were mild or moderate in severity. The incidence of cheek-specific AEs was comparable between the autologous fibroblast– and vehicle control–treated cheeks. The most common AEs were treatment area erythema (occurring in 11.1% of subjects) and swelling (occurring in 10.1% of subjects). Of the related treatment area AEs occurring in more that 5% of subjects, all were of mild or moderate severity (Table 5), and 89% of all treatment area AEs resolved within 1 week. Five of the 12 subjects reporting erythema and five of the 11 subjects reporting swelling had events of moderate severity in the autologous fibroblast–treated area, whereas all 11 related AEs reported in the vehicle control–treated area were of mild severity. Five subjects experienced erythema and swelling at both treatment areas, where the AE was of moderate severity on the cheek receiving active treatment. The subjects received cold compress therapy for these AEs, which resolved in 1 to 4 days. No clinically meaningful changes in skin pigmentation or evidence of hypertrophic scarring in the treated areas was observed. No changes in vital signs or physical examination findings were reported. Of the subjects who completed the study, 98% indicated that they were interested in receiving additional treatment with autologous fibroblast.

**Table 5:** Related Treatment Emergent Adverse Events by Severity Reported in >5% Subjects

*Adverse Event Severity*	*Autologous Fibroblast, n = 99*	*Vehicle Control, n = 99*
	N (%)		
Erythema		
Mild	7 (7)	11 (11)
Moderate	5 (5)	0
Swelling		
Mild	6 (6)	11 (11)
Moderate	5 (5)	0

Treatment Emergent Adverse Events preferred terms were defined using the Medical Dictionary for Regulatory Activities version 10.0. The table shows the number and percentage of subjects that experienced treatment-emergent adverse events according to severity and considered by the investigator to be related (possibly, probably, definitely) to study treatment. Both adverse events were located at the treatment area.

## Discussion

Autologous fibroblast treatment is a novel natural method for correcting dermal defects that involves in vivo injection of autologous fibroblasts into contour defects. Autologous fibroblasts have the ability to produce human collagen in vivo, which obviates the need for skin testing previously required for use of products containing bovine collagen. The mechanism of action of autologous fibroblast treatment is not well understood, but previous clinical results suggest that novel collagen production and remodeling of preexisting extracellular matrix in the scarred tissue may be associated with the observed improvement.^20^ A previous placebo-controlled clinical trial evaluating injections of autologous fibroblasts for the treatment of facial contour deformities found significantly greater improvements in acne scar appearance than with placebo in the subset of subjects with facial acne scarring.^20^ To some extent, these scars are a result of a loss of collagen after resolution of localized intense inflammation and wound healing associated with inflammatory acne. Injection of inert filler materials temporarily corrects the tissue defect, but the biologic environment is unchanged. Consequently, scar appearance typically reverts to its pretreatment appearance once the inert material is resorbed. Autologous fibroblast treatment offers one method of correcting the biologic elements supporting scar morphology, which could lead to better treatment outcomes. Earlier studies have demonstrated objective and subjective diminution of contour facial defects after treatment with autologous fibroblasts.^21^–^22^ Histologic analysis demonstrated that areas treated with fibroblast injections had a thicker, denser layer of collagen in the dermal region, showed no evidence of inflammatory reaction, and contained viable fibroblasts throughout.^22^

Treatment with autologous fibroblast was associated with statistically significantly greater improvement in acne scar appearance than vehicle control in the treatment of moderate to severe acne scarring based on the live subject and evaluator responder analysis and three independent photographic reviewer assessments. The subjects in this study tended to have more-extensive distensible acne scarring than the population typically treated in a dermatologist’s office because of the inclusion criteria. Although the difference in the percentage of responders between autologous fibroblast and vehicle control treatment for the evaluator and the subject responder analysis was similar (∼20%), evaluators rated more vehicle-treated cheeks as responders (42.2%) than did subjects (18.3%). Despite the magnitude of the placebo effect associated with the vehicle control treatment in the evaluator responder analysis, the autologous fibroblast–treated cheek was associated with a statistically significantly greater (58.7%) number of responders (*p* = .01). The association between the vehicle control treatment and a significant treatment response is not unexpected given that techniques such as skin needling and subcision, which are believed to disrupt collagen fibers that anchor the superficial dermis to dermal and subdermal layers and stimulate collagen synthesis, are used to treat acne scarring.^23^–^24^ Given that the responder definitions differed between the subject and evaluator assessments (subject assessments required a 2-point improvement from baseline, whereas the evaluator assessment required a 1-point improvement), it is not unexpected that the evaluator assessment had more responders for the active and vehicle control treatments (64 and 46 cheeks, respectively) than the subject assessment responders (47 and 20 cheeks, respectively). The live assessments identified a greater magnitude of improvement in acne scar appearance than did the IPR assessments, although the internal consistency in the efficacy results is notable given that the primary efficacy assessments were based on live assessments with no baseline comparator, whereas the IPR results were based on comparison of pairs of photographs.

Quantifying acne scarring and changes in acne scars after an intervention is particularly difficult given the diverse types of scarring. To address this challenge, a validated, novel, 5-point acne scar severity assessment and accompanying photo guide were used for this study for the live assessment. Depressed, distensible facial acne scars treated in this study were classified as an atrophic subset of the Grade 3 category according to Goodman and Baron.^2^ Validation of the evaluator acne scar assessment scale demonstrated that 1-point improvements on this scale, as judged by trained evaluators, are reliable third-party indicators of positive treatment effect, providing evidence of clinically meaningful improvement in acne scarring appearance after autologous fibroblast treatment. Subject assessment of acne scar appearance is arguably the most important assessment of treatment outcome because the subjects are the beneficiaries of the treatment.^25^ No validation studies of a 5-point subject assessment of satisfaction scale have been published, but using a similar 5-point classification system with two categories indicating positive change (e.g., satisfaction), one category for no change, and two categories for negative change, a 1-point change was defined as a minimal clinically important difference when the 5-point scale was used to determine the minimal clinically important difference for a novel acne-specific 15-point scale.^26^ Thus the definition of a responder in this study (2-point improvement on the subject assessment and 1-point improvement on the evaluator assessment) probably exceeds what would be considered a minimal clinically important difference in acne scar appearance after autologous fibroblast treatment of depressed distensible acne scars.

**Figure 4:**
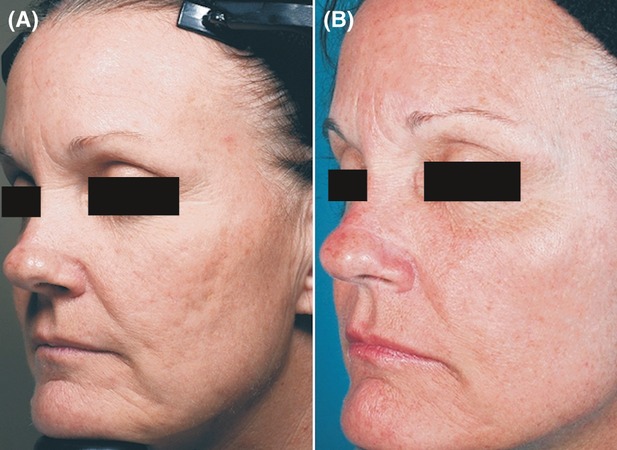
Sustained response noted from subject on the autologous fibroblast–treated left cheek 2 years after the last study treatment: (A) baseline, (B) 2 years after treatment (54 × 39 mm; 300 × 300 dots per inch).

**Figure 5:**
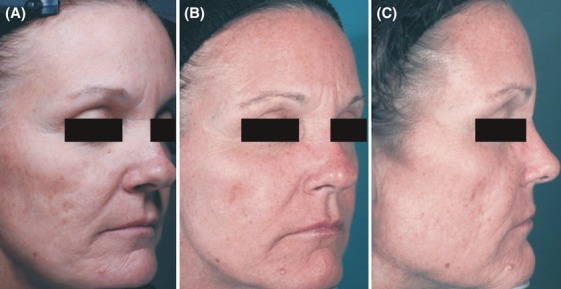
Sustained response from subject BJ on the control side right cheek 2 years after the last study treatment: (A) baseline, (B and C) 2 years after treatment (54 × 27 mm; 300 × 300 dots per inch).

**Figure 6:**
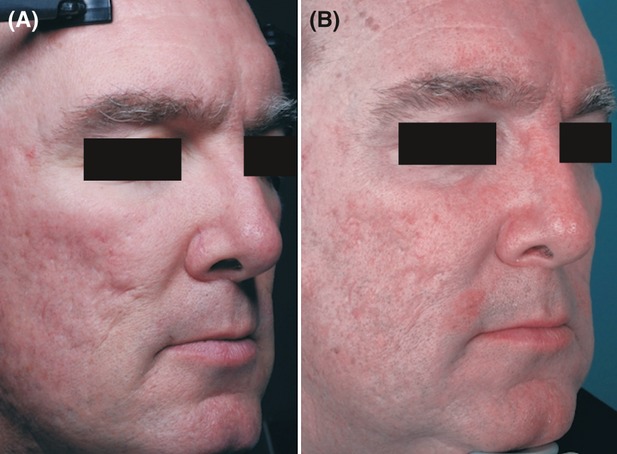
Sustained response from autologous fibroblast–treated cheek on study subject 2.5 years after final study treatment: (A) baseline, (B) 2.5 years after treatment (54 × 39 mm; 300 × 300 dots per inch).

It it is likely that autologous fibroblast treatment is associated with a durable response because anecdotal reports from subjects treated for scars with autologous fibroblasts in a previous study suggest that the duration of efficacy may be long lasting. Specifically, 75% to 82% of autologous fibroblast–treated subjects continued to demonstrate treatment benefit 9 and 12 months after treatment.^20^ Additionally, anecdotal 2-year follow-up of several subjects from one site showed persistent improvement in acne scar appearance. Figures 4 and 5 represent 2-year follow-up from one study site. Sustained improvement in the appearance of acne scarring (two years post last treatment) is depicted on the treated left cheek (Figure 4) versus the control right cheek (Figure 5) (Munavalli, Charlotte, NC). Another subject is depicted in Figure 6 with sustained improvement 2.5 years after the last study treatment.

Based on the nature of the treatment-emergent AEs reported, autologous fibroblasts were safe and well-tolerated in this study. The split-face study design clearly indicated that the incidence of treatment-emergent AEs was comparable on the autologous fibroblast– and vehicle control–treated sides of the face, and the types of observed AEs were not unexpected given the 25 to 40 intradermal injections per side per treatment. As might be expected, treatment area erythema and swelling was somewhat more pronounced on autologous fibroblast–treated cheeks (more events rated moderate) than on vehicle-treated cheeks, but there was no difference in the duration of treatment-emergent AEs between the two treatment areas.

Unlike acne scarring treatment using ablative laser resurfacing, fractional nonablative resurfacing^2^–^15^ or other less-invasive treatment options such as subcision, autologous fibroblast treatment is associated with a minimal post-treatment recovery period and no post-treatment purpura or pigmentary alteration, even in subjects with Fitzpatrick skin types IV and V in this study. No subjects discontinued treatment with autologous fibroblasts as a result of treatment discomfort or AEs, and subjects who experienced AEs returned for retreatment, indicating the mild nature of the events.

In summary, autologous fibroblast treatment was associated with clinically meaningful improvement in acne scar appearance and a positive risk:benefit ratio, as reflected by subjects’ continuing interest in participation in a future study. Autologous fibroblast treatment was safe and superior to vehicle control for the treatment of moderate to severe depressed distensible facial acne scarring.
